# The Effects of *Lactococcus garvieae* and *Pediococcus pentosaceus* on the Characteristics and Microbial Community of *Urtica cannabina* Silage

**DOI:** 10.3390/microorganisms13071453

**Published:** 2025-06-23

**Authors:** Yongcheng Chen, Shuangming Li, Yingchao Sun, Yuxin Chai, Shuan Jia, Chunhui Ma, Fanfan Zhang

**Affiliations:** College of Animal Science and Technology, Shihezi University, Shihezi 832000, China; chenyonchn@163.com (Y.C.);

**Keywords:** nettle silage, lactobacillus inoculation, microbial community regulation, feed resource development

## Abstract

The utilization of nettle (*Urtica cannabina*) as feed is restricted by its material properties (antibacterial activity and high buffering capacity). This study hypothesized that the use of lactic acid bacteria (LAB) attached to nettles can improve these problems. *Lactococcus garvieae* (LG), *Pediococcus pentosaceus* (PP), and LG + PP (LP) isolated from nettles were inoculated into nettle silage to explore nutrient retention and the microbial community structure. The results showed that inoculation significantly delayed dry matter and crude protein loss, inhibited neutral detergent fiber and acid detergent fiber degradation, and reduced ammonia nitrogen (NH_3_-N) accumulation. There was a significant increase in Firmicutes abundance after inoculation, and the dominant genus, *Aerococcus,* was negatively correlated with NH_3_-N accumulation. In the later stages of the PP treatment, *Atopistipes* synergistically inhibited *Clostridia* with acetic acid. However, the high buffering capacity and antibacterial components of raw nettle led to increased pH values during the later fermentation stages, limiting sustained acid production by LAB. These results confirm that nettle-derived LAB can effectively improve the quality of silage by regulating the microbial community and the acidification process; however, they must be combined with pretreatment strategies or optimized composite microbial agents to overcome raw material limitations. This study provides a theoretical basis and technical support for the utilization of nettle as feed.

## 1. Introduction

Nettle (*Urtica cannabina*) is a perennial herb found in the genus *Urtica*. Nettles are widely distributed worldwide in regions with mild to temperate climates, including North Africa and parts of Asia, Europe, and North America [1]. In China, nettles are mainly distributed in temperate regions, such as Xinjiang, Gansu, Sichuan, and Shaanxi [2]. Currently, there is a large gap in the feed supply, and the problem of feed shortages must be addressed urgently. Nettles have a wide distribution range, a high yield, and a high nutritional value, and they possess great development potential as a feed resource [3]. Moreover, nettles contain various bioactive substances that endow them with anti-inflammatory and antioxidant properties, and animals can effectively improve their immunity after consuming nettles [4,5]. Adding microbial inoculants for silage preparation is the primary method of long-term feed preservation [6]. The selection of lactic acid bacteria (LAB) inoculants directly affects the success of silage, as well as its nutritional and fermentation quality [7]. Common LAB genera in silage include *Pediococcus*, *Enterococcus*, *Lactococcus*, and *Lactobacillus*—all of which are homofermentative LAB [8]. These bacteria utilize soluble carbohydrates through the Embden–Meyerhof–Parnas glycolytic pathway to produce large amounts of lactic acid. They rapidly consume fermentation substrates to grow and reproduce in the early stages of silage, producing substantial lactic acid quantities, thereby reducing the pH of the fermentation system and dominating the fermentation process [9]. Lactic acid bacteria can be classified into endogenous (naturally occurring) and exogenous (externally inoculated) strains according to their sources [10]. Owing to the characteristics of raw materials and environmental conditions, using LAB strains naturally attached to a raw material may improve the quality and reliability of silage fermentation [11,12].

As a highly valuable unconventional feed, nettle leaves possess high nutritional and medicinal value; however, current processing methods are limited to fresh feeding and dry hay production [13]. Although nettle is rich in nutrients and bioactive compounds, it presents specific challenges during ensiling owing to its inherent antibacterial activity and high buffering capacity. In previous studies, the fermentation and nutritional quality of nettle silage inoculated with commercial LAB strains were poor. Improved results were observed only when formic acid or corn flour was added to the silage [14]. Our earlier attempts to ferment nettle silage using commercial inoculants failed to achieve a pH < 4.5 and were accompanied by an increase in the ammonia nitrogen to total nitrogen ratio (NH_3_-N/TN) and a reduction in the water-soluble carbohydrate (WSC) content, indicating that conventional laboratory strains lack adaptability to the stressors unique to nettle [5].

Therefore, in this study, two LAB strains with excellent acid-producing and growth characteristics were isolated from microorganisms naturally present on nettle. *Lactococcus garvieae* and *Pediococcus pentosaceus* were inoculated into nettle silage individually and in combination. Nutritional and fermentation quality indicators were assessed. We hypothesized that these two strains could overcome nettle’s antibacterial barrier by utilizing fiber degradation products to sustain acid production after initial WSC depletion. We aimed to elucidate how the interaction between the two strains regulates microbial communities and whether their metabolic byproducts can mitigate pH rebound during the later fermentation stages. Addressing these issues established a framework for developing inoculants adapted to nettle, thereby providing a foundation for future research and the utilization of nettle as a plant-based feed resource.

## 2. Materials and Methods

### 2.1. Experimental Materials

Nettle (*Urtica cannabinae*) was collected from the stone field of Bortonggu pasture in Shawan City, Tacheng District, Xinjiang Uygur Autonomous Region (84°58′–86°24′ E; 43°26′–45°20′ N). Harvesting was performed manually on 20 September 2023 (autumn). The plant height was about 90 cm, and the plant parts 5 cm above the ground were collected. This region belongs to a continental temperate arid climate, with an average temperature of 6.3 to 6.9 °C. The annual sunshine duration is 2800–2870 h, with a cumulative temperature of 3400 to 3600 °C above 10 °C. The frost-free period is 170–190 d, with an annual precipitation of 140 to 350 mm and an annual evaporation of 1500 to 2000 mm. We screened two strains of LAB as silage additives in our previous experiment on nettle silage, namely *Lactococcus garvieae* (GenBank number: OL454842) and *Pediococcus pentosaceus* (GenBank number: OL45560411) [15]. The strains were cultured in a De Man, Rogosa, and Sharpe (MRS) broth medium for 24 h: *Lactococcus garvieae* (pH = 3.82, OD_600_ = 2.12) and *Pediococcus pentosaceus* (pH = 3.78, OD_600_ = 2.06). The LAB were removed from a −80 °C freezer, activated, and cultured on an MRS agar medium for three generations before being transferred to the MRS broth medium for further cultivation. The quantity was determined by plate counting (the coating method) [16].

### 2.2. Experimental Design

The collected fresh nettles were transferred with a fresh weight of approximately 300 g/kg, crushed immediately to 1–2 cm, and mixed well, and approximately 1 kg of raw nettle material was weighed and placed in a polyethylene bag (23 cm × 40 cm) with a one-way vacuum suction valve. This experiment adopted a two-factor (inoculation and fermentation time) completely randomized design. The LG treatment was inoculated with *Lactococcus garvieae* (5 × 10^6^ cfu/g); the PP treatment was inoculated with *Pediococcus pentosaceus* (5 × 10^6^ cfu/g); the LP treatment was inoculated with both *Lactococcus garvieae* and *Pediococcus pentosaceus* (5 × 10^6^ cfu/g, inoculation ratio: 1:1) [17,18]; and an equal amount of sterile water was added to the control group (CK). Thirty replicates were prepared per treatment. After ensiling, the products were stored and fermented at room temperature. Destructive sampling was performed on five randomly chosen replicates per treatment at specified time points (0, 7, 15, 30, and 60 d post-ensiling) during ensiling for nutritional and fermentation quality testing.

### 2.3. Characteristics of Wilted and Ensiled Nettle

The raw nettle materials and silage samples were heated at 105 °C for 2 h, then transferred to 65 °C to reach a constant weight, and the dry matter (DM) content was calculated. The samples were crushed using a plant pulverizer (DFY-1000D, Zhejiang Wenling Linda Instrument Co., Ltd., Wenling, China) and passed through a 0.42 mm sieve for subsequent analysis. The crude protein (CP) content was measured using an automatic Kjeldahl nitrogen analyzer (K-375, Buchi, Uster, Switzerland), and the CP was calculated according to the method of the Association of Official Agricultural Chemists (AOAC). The neutral detergent fiber (NDF) and acid detergent fiber (ADF) contents were measured using a fully automated fiber analyzer (ANKOM A200i, ANKOM Technology, Macedon, NY, USA). Ether extract (EE) was analyzed using a fully automated fat analyzer (SOX416, Gerhardt, Königswinter, Germany). Water-soluble carbohydrates (WSCs) were determined using the anthrone colorimetric method (722 Visible Spectrophotometer, Shanghai Precision Technology Instrument Co., Ltd., Shanghai, China) [16]. A mixture of silage and water (*v*/*v* = 1:9) was placed in a refrigerator at 4 °C for 24 h, filtered, and centrifuged. The pH was measured using a pH meter (PHS-3C, Shanghai Instrument and Electrical Science Instrument Co., Ltd., Shanghai, China), and the supernatant was collected for analysis of the ammonia nitrogen to total nitrogen ratio (NH_3_-N/TN). Organic acid (lactic acid, LA; acetic acid, AA; propionic acid, PA; and butyric acid, BA) concentrations were measured using a C18 column for high-performance liquid chromatography (HPLC). Na_2_HPO_4_ (1 mM, pH 2.7) and methanol were used for mobile phases A and B, respectively, with a flow rate of 0.6 mL/min and an injection volume of 20 μL according to Weatherburn (1967) [19]. Five duplicate data were obtained for each processing group.

### 2.4. Bacterial Community Analysis

Deoxyribonucleic Acid (DNA) was extracted from feed samples using an E.Z.N.A.^®^ Soil Kit (Omega Bio-tek, Norcross, GA, USA) (three replicates for each treatment group). The quality of the DNA was assessed using 1% agarose gel electrophoresis, and its concentration and purity were determined using a microvolume spectrophotometer (NanoDrop One, Thermo Fisher Scientific, Waltham, MA, USA). The V3–V4 variable region of the 16S rRNA gene was amplified using the Polymerase Chain Reaction (PCR), with DNA extracted from the feed samples as the template. The primers used were 338F (5′-ACTCCTACGGGAGGCAGCAG-3′) and 806R (5′-GGACTACHVGGGTGTCTAAT-3′). PCR products were recovered and detected using 2% agarose gel electrophoresis. A sequencing library was constructed from purified PCR products and sequenced on the Illumina platform, followed by high-throughput sequencing data analysis [20,21].

### 2.5. Statistical Analysis

Raw data was gathered and prepared for statistical analysis using Microsoft Office Excel 2021. Data normality was confirmed by the Shapiro–Wilk test, and homogeneity of variance was verified using Levene’s test. A two-way ANOVA was adopted (IBM SPSS version 17.0; SPSS Inc., Chicago, IL, USA). When the interaction effect was significant, simple effect analysis of a single-factor ANOVA with separate fixations was conducted. Differences between treatments were considered significant by Duncan’s test at *p* < 0.05. The data are presented as the average values and the standard deviations. The sequencing data of the bacterial communities were analyzed using the free online Majorbio Cloud Platform (www.Majorbio.com), accessed on 20 February 2025. The Shannon, Sobs, and Chao 1 diversity indices were calculated through the boot (1.3.18) and stats (3.3.1) packages of the R language (version 3.3.1), while the β diversity was analyzed using principal coordinate analysis (PCoA) and tested by ANOSIM with the vegan package in R using the Bray–Curtis distance algorithm. The Spearman method was used to conduct correlation analysis on the bacterial communities and main fermentation tables in different periods.

## 3. Results

### 3.1. Nutritional Quality and Fermentation Characteristics of Nettle Silage

Changes in the nutritional quality and fermentation characteristics of nettle silage during fermentation are shown in Table 1. The results show that the DM, CP, NDF, ADF, EE, and WSC contents in each treatment decreased with increasing fermentation time. From the perspective of the overall fermentation process (0–60 days), the loss of most nutrients (DM, CP, NDF, ADF, EE, and WSCs) mainly occurred in the early stage of fermentation (such as within the first 15 days or 30 days), and the downward trend tended to level off or the variation range decreased in the later stage (30–60 days). The inoculation treatments (LG, PP, and LP) were superior to CK, and the LG treatment most significantly slowed the decrease in DM (*p* < 0.05). The decrease in CP in the LG and PP treatments was relatively low. At 30 d of ensiling, there was no significant difference in the NDF content between CK and the inoculation treatments (*p* > 0.05). However, on the 60th d of fermentation, the NDF content in the inoculation treatments was significantly higher than that in CK (*p* < 0.05). The ADF content in the LG treatment was significantly higher than that in the other treatments (*p* < 0.05). The EE and WSC contents decreased significantly with prolonged fermentation time, and on the 60th d of fermentation, the EE content in the LG and PP treatments was significantly higher than that in CK (*p* < 0.05); however, there was no significant difference in the WSCs between the treatments on the same fermentation days (*p* > 0.05).

Through the changes in the fermentation characteristics of the nettle silage during fermentation (Table 2), we know that with extension of the fermentation time, the pH of the inoculation treatments showed a decreasing trend, whereas the LA content showed an increasing trend. The content changes of LA, AA, and PA showed obvious phased characteristics. The contents of AA and PA increased on the 7th and 15th d of fermentation but decreased on the 30th and 60th d. The NH_3_-N/TN ratio in the LG, PP, and LP treatments was significantly lower than that in CK, but the NH_3_-N/TN of all treatments increased significantly on the 30th and 60th d of fermentation (*p* < 0.05). The pH of CK increased on the 30th and 60th d of fermentation, whereas the LA, AA, and PA contents first increased and then decreased. The pH values of the LG and PP treatments were significantly lower than those of CK and the LP treatment on fermentation days 15, 30, and 60 (*p* < 0.05). The LA and AA contents of the LG, PP, and LP treatments were significantly higher than those of CK on the 30th and 60th d (*p* < 0.05). The PA content of the LP treatment was significantly higher than that of the other treatments on the 15th d of fermentation (*p* < 0.05). The BA content in CK was significantly higher than that in the other treatments on the 60th d of fermentation (*p* < 0.05). On the 7th, 15th, and 60th d of fermentation, the NH_3_-N/TN content in the LG treatment was significantly lower than that in the other treatments (*p* < 0.05).

### 3.2. Alpha Diversity Analysis of Nettle Silage and Beta Diversity Analysis

**Figure 1:**
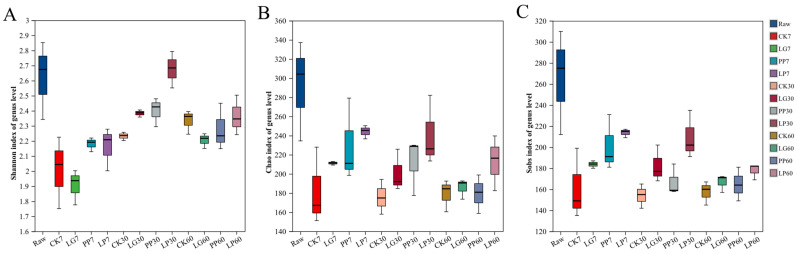
Alpha diversity analysis of nettle silage: Shannon index (**A**), Chao index (**B**), and Sobs index (**C**). Raw, raw material; CK, control treatment; LG, inoculation with *Lactococcus garvieae*; PP, inoculation with *Pediococcus pentosaceus*; LP, co-inoculation with *Lactococcus garvieae* and *Pediococcus pentosaceus*. Labels with 7, 30, and 60 represent 7th, 30th, and 60th days, respectively.

Microbial community data were acquired through high-throughput sequencing of nettle silage samples fermented for 7, 30, or 60 d. The sequencing depth was adequate, and 2,608,811 optimized sequences were obtained. The average effective sequence length of 422 bp encompassed most of the species information within the microbial community. At a similarity threshold of 97%, all sequenced reads were clustered into 2512 operational taxonomic units. Microbial diversity analysis revealed notable differences between the treatment groups. Figure 1A–C show the dynamics of the Shannon, Sobs, and Chao indices. As shown in the figure, the inoculation treatment had a significant effect on the microbial diversity of the nettle silage. The diversity and richness of the LP treatment were significantly higher than those of the other treatments at 30 d (*p* < 0.05). From the perspective of fermentation time, the diversity and richness of all treatment groups were generally higher at 30 d than at 7 or 60 d.

**Figure 2:**
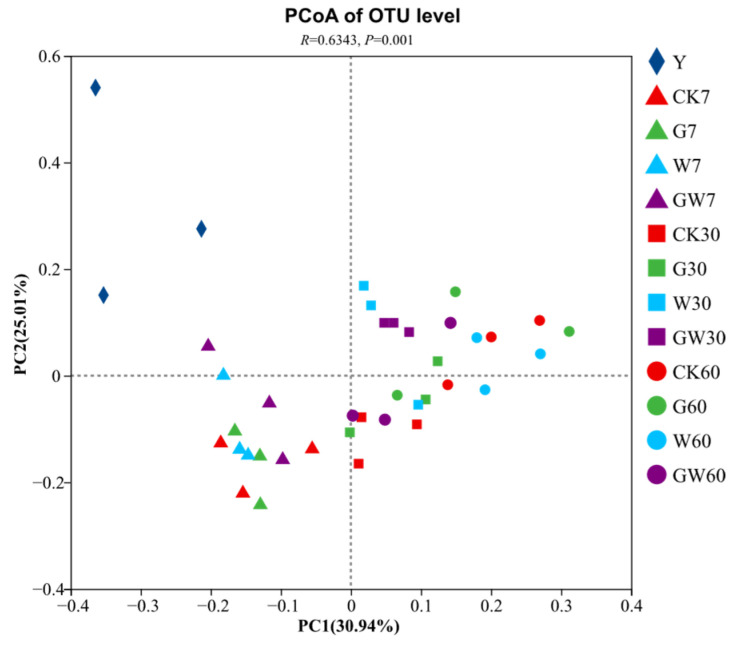
Principal coordinate analysis (PCoA) plots for the bacterial community in the nettle silage. OTU, operational taxonomic unit. Raw, raw material; CK, control treatment; LG, inoculation with *Lactococcus garvieae*; PP, inoculation with *Pediococcus pentosaceus*; LP, co-inoculation with *Lactococcus garvieae* and *Pediococcus pentosaceus*. Labels with 7, 30, and 60 represent the 7th, 30th, and 60th days, respectively.

As illustrated in Figure 2, principal coordinate analysis was used to evaluate the bacterial communities in the raw materials (Raw), the control treatment (CK), the treatment inoculated with *Lactococcus garvieae* (LG), the treatment inoculated with *Pediococcus pentosaceus* (PP), and the treatment co-inoculated with both strains (LP). The results show that, compared with CK, the inoculation treatments altered the distribution of the bacterial communities, but there were no significant differences among the inoculation treatments over time (R = 0.6343, *p* < 0.01).

### 3.3. Analysis of Microbial Community Composition of LAB Inoculated with Urtica cannabinae

**Figure 3:**
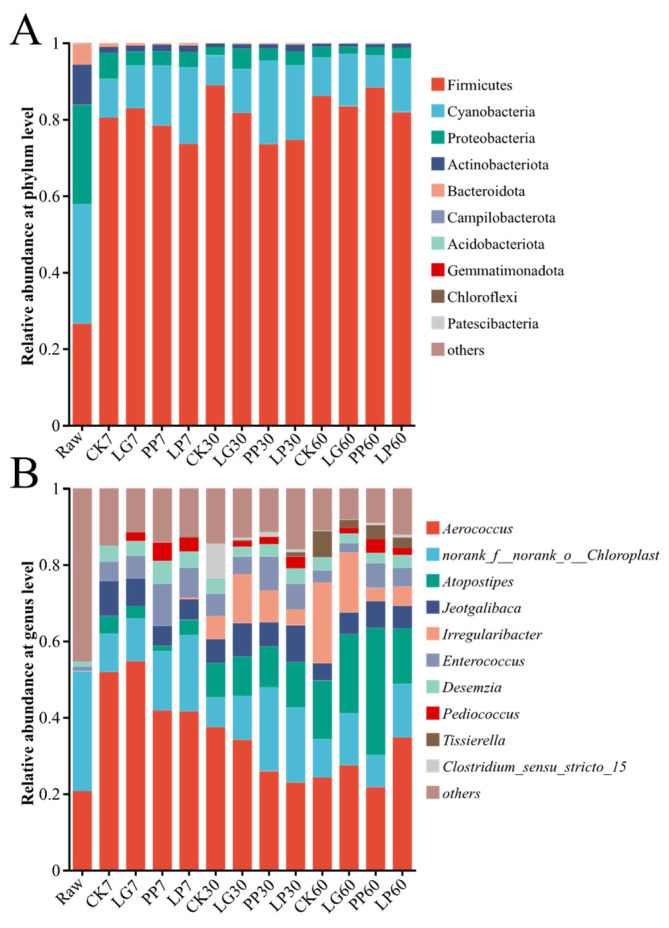
Bacterial community and relative abundances of nettle silage based on phyla (**A**) and genera (**B**). Raw, raw material; CK, control treatment; LG, inoculation with *Lactococcus garvieae*; PP, inoculation with *Pediococcus pentosaceus*; LP, co-inoculation with *Lactococcus garvieae* and *Pediococcus pentosaceus*. Labels with 7, 30, and 60 represent the 7th, 30th, and 60th days, respectively.

Figure 3A illustrates the dynamic shifts in the relative abundances of phylum-level microbial communities in the raw materials and various treatment groups after 7, 30, and 60 d of fermentation. Among the raw materials, Cyanobacteria, Firmicutes, Proteobacteria, Actinobacteria, and Bacteroidetes collectively accounted for more than 99% of the total microbial abundance. Upon fermentation initiation, Firmicutes rapidly became dominant across all treatments (73.51–88.93%), followed by Cyanobacteria (7.82–21.92%) and Proteobacteria (1.86–6.89%). Cyanobacteria, Proteobacteria, Actinobacteria, and Bacteroidetes exhibited treatment- and duration-dependent variations but showed overall downward trends relative to the raw materials (*p* < 0.05). Specifically, at 7 d, the Cyanobacteria (10.20–20.04%) and Proteobacteria (3.56–6.89%) abundances declined in all treatments (*p* < 0.05). However, by 30 d, the PP treatment displayed a 6.32% increase in Cyanobacteria abundance, whereas the LG treatment showed a 1.80% increase in Proteobacteria abundance. After 60 d of fermentation, the relative abundances of Firmicutes in the PP and LG treatments further increased to 88.32% and 83.45% (*p* < 0.05), respectively, compared with their respective abundances at 30 d. By contrast, the CK and LP treatments demonstrated slight reductions in Firmicutes dominance, revealing treatment-specific divergence in the microbial community structure.

Figure 3B shows the genus-level temporal succession of the microbial communities during nettle silage fermentation. The raw material was dominated by the unclassified taxon *norank_f__norank_o__Chloroplast* spp. (31.28%) and *Aerococcus* spp. (20.76%). After 7 d of fermentation, *Aerococcus* spp. exhibited a marked increase in relative abundance across the treatments (CK, 51.92%; LG, 54.75%; PP, 41.82%; and LP, 41.58%) (*p* < 0.05). Although their abundance declined slightly by 30 d, *Aerococcus* spp. remained the predominant genus in all treatments. At the 60 d endpoint, *Atopostipes* spp. surpassed *Aerococcus* spp. (21.75%) as the dominant taxon in the PP treatment, achieving an abundance of 33.24% (*p* < 0.05). By contrast, *Aerococcus* spp. retained dominance in the other treatments, whereas *Atopostipes* spp. concurrently demonstrated significant enrichment (14.57–21.19%) (*p* < 0.05), ultimately establishing a stable symbiotic multi-genus structure.

**Figure 4:**
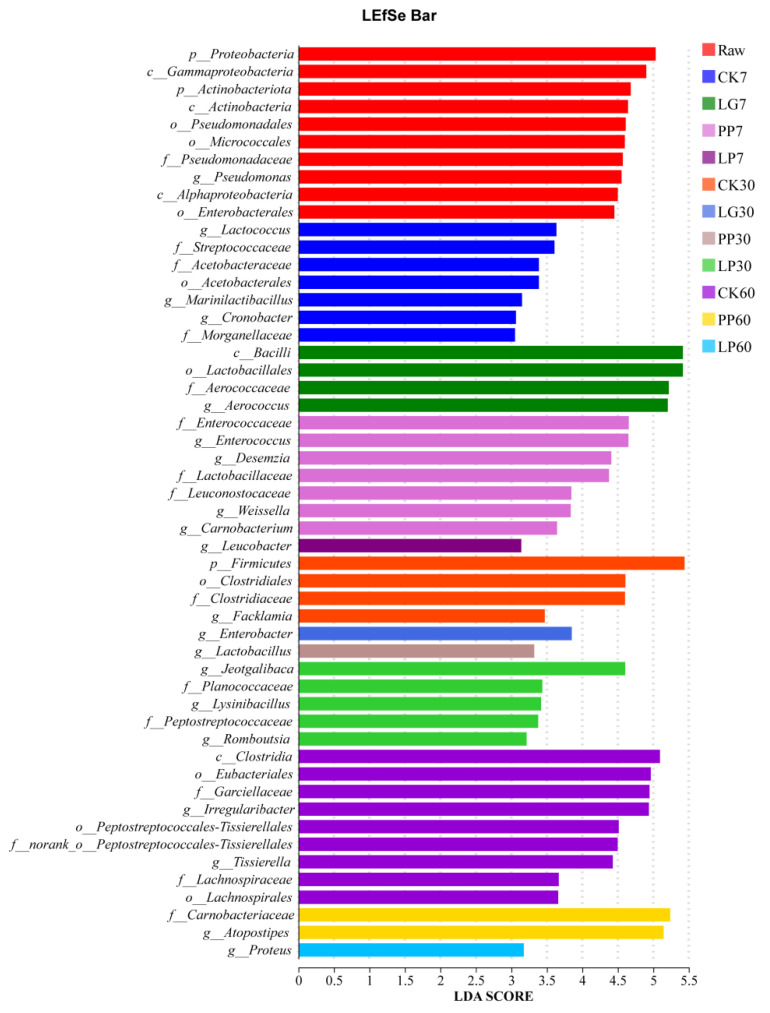
Comparison of microbial variations using LEfSe analysis of nettle silage. Raw, raw material; CK, control treatment; LG, inoculation with *Lactococcus garvieae*; PP, inoculation with *Pediococcus pentosaceus*; LP, co-inoculation with *Lactococcus garvieae* and *Pediococcus pentosaceus*. Labels with 7, 30, and 60 represent the 7th, 30th, and 60th days, respectively.

Figure 4 shows the signature microbial taxa across the different treatments and time points during nettle silage fermentation using the linear discriminant analysis effect size (LEfSe analysis). In the CK treatment, *Lactococcus* spp. and *Streptococcaceae* spp. served as signature taxa during the early fermentation stage (7 d). *Aerococcus* spp. were identified as the key discriminative taxon in the LG treatment, whereas *Pediococcus* spp. and *Weissella* spp. were aligned with the initial community restructuring in the PP treatment. By 30 d, the LG treatment exhibited significant enrichment of *Pedobacter* spp. and *Jeotgalibaca* spp. (*p* < 0.05), whereas *Lactobacillus* spp. emerged as a divergent taxon in the PP treatment. At the 60 d mark, *Aerococcus* spp. (in the LG and LP treatments) and *Atopostipes* spp. (in the PP treatment) retained their persistent dominance (*p* < 0.05), maintaining both functional relevance and superior abundance.

**Figure 5:**
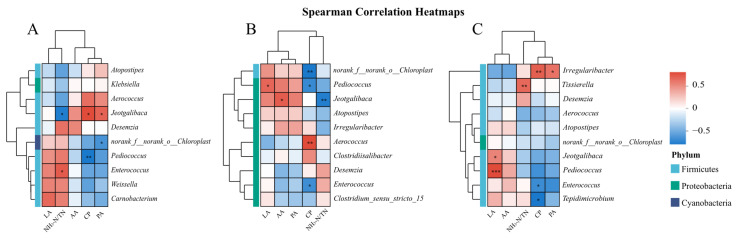
Correlation analysis heatmaps showing associations between the abundances of bacterial genera and the fermentation characteristics of nettle silage at 7 d (**A**), 30 d (**B**), and 60 d (**C**). * *p* < 0.05, ** 0.01 < *p* < 0.05, *** *p* < 0.01. AA, acetic acid; LA, lactic acid; PA, propionic acid; CP, crude protein.

Figure 5 presents heatmaps showing the Spearman correlations between the variance inflation factor (VIF)-screened fermentation parameters (LA, AA, PA, CP, and NH_3_-N/TN) and the top 10 microbial genera during nettle silage fermentation at 7 d (5A), 30 d (5B), and 60 d (5C). At 7 d, NH_3_-N/TN accumulation showed a significant negative correlation with *Jeotgalibaca* spp. (*R* = –0.69, *p* < 0.05) and a positive correlation with *Enterococcus* spp. (*R* = 0.61, *p* < 0.05). CP (*R* = 0.67, *p* < 0.05) and PA (*R* = 0.58, *p* < 0.05) were positively correlated with *Jeotgalibaca* spp., whereas CP exhibited a strong negative correlation with *Pediococcus* spp. (*R* = –0.72, *p* < 0.01). At 30 d, CP was negatively correlated with *norank_f__norank_o__Chloroplast* spp. (*R* = –0.76, *p* < 0.01) and *Pediococcus* spp. (*R* = –0.67, *p* < 0.05), whereas LA was positively correlated with *Pediococcus* spp. (*R* = 0.63, *p* < 0.05). *Jeotgalibaca* spp. displayed opposing associations with AA (*R* = 0.68, *p* < 0.05) and NH_3_-N/TN (*R* = –0.73, *p* < 0.01). CP showed divergent correlations with *Aerococcus* spp. (*R* = 0.75, *p* < 0.01) and *Enterococcus* spp. (*R* = –0.64, *p* < 0.05). At 60 d, *Irregularibacter* spp. were positively correlated with CP (*R* = 0.75, *p* < 0.01) and PA (*R* = 0.67, *p* < 0.05), whereas *Tissierella* spp. (*R* = 0.71, *p* < 0.01) were linked to NH_3_-N/TN accumulation. LA exhibited a strong positive correlation with *Jeotgalibaca* spp. (*R* = 0.60, *p* < 0.05) and *Pediococcus* spp. (*R* = 0.84, *p* < 0.001). CP was negatively associated with *Enterococcus* spp. (*R* = –0.68, *p* < 0.05) and *Tepidimicrobium* spp. (*R* = –0.58, *p* < 0.05).

## 4. Discussion

### 4.1. Effects of Inoculating LAB from Urtica cannabinae on Nutritional and Fermentation Quality of Nettle Silage

Loss of DM during the silage process is mainly caused by microbial metabolic consumption of the substrate [7]. Inoculation with LAB (LG, PP, and LP) significantly slowed the decline in DM by rapidly establishing dominant flora, and the LG treatment showed the best retention of DM. As shown in Figure 3B, the abundance of *Aerococcus* spp. increased significantly in the inoculated treatments, which was closely related to the ability of LAB to reduce the activity of aerobic bacteria, molds, and yeasts through competitive inhibition [22,23]. Loss of CP was minimal in the early stage of fermentation but increased during the later stage, a trend associated with the reduced abundance of Firmicutes in CK and increased activity of cellulolytic bacteria, such as Bacteroidetes [24]. Simultaneously, the increase in NH_3_-N/TN indicated that *Clostridia* spp. contributed to protein decomposition, whereas inoculation suppressed the metabolism of such microorganisms by maintaining a low pH [25].

The inoculated silages retained significantly higher NDF and ADF contents than CK at 60 d, whereas no differences between treatments were detected at 30 d, confirming time-dependent lignocellulose degradation [26,27]. This may be attributed to enhanced activity of cellulose-degrading bacteria under un-inoculated conditions [17,28]. EE preservation was 10–15% greater in the inoculated groups, likely because of inhibition of lipolytic microorganisms by LAB [29]. Rapid consumption of WSCs (a 50% reduction within 7 d) reflected competitive utilization of carbon sources by LAB in the early stage. Fiber transformation in the later stage may provide alternative carbon sources for microorganisms [30,31], although the WSC levels showed no treatment-specific differentiation.

The pH trend indicated that although the inoculated treatments maintained a significantly lower pH than CK, the overall decline was modest (only 0.5 units in 7 d), likely because of the high buffering capacity of nettles [5,29]. Nevertheless, the pH in the inoculated treatments ultimately reached the range indicative of high-quality silage (4.2–4.5). LEfSe analysis revealed transient *Lactococcus* spp. dominance in CK that collapsed after 15 d, suggesting that antimicrobial phytochemicals in nettle may inhibit LAB proliferation [5,22]—a hypothesis warranting further validation via metabolomic analysis.

During inoculation, the LA content increased initially and then decreased with fermentation time, peaking at 30 d. LA was subsequently decomposed by *Clostridia* spp. at 60 d, likely owing to a pH increase, as evidenced by the significant rise in the BA content in CK. The synergistic effect of AA and LA was more pronounced in the inoculated treatments, especially in PP, where Lactobacillus abundance was positively correlated with AA accumulation (R = 0.68), further inhibiting *Clostridia* spp. activity [5,32].

### 4.2. Effect of Inoculating LAB Derived from Urtica cannabinae on Composition of Nettle Silage Microbiota

The dynamic succession of microbial communities during silage fermentation is closely associated with nutrient metabolism [8]. High-throughput sequencing analysis revealed that inoculation with LAB (LG, PP, and LP) significantly increased the relative abundance of Firmicutes (73.51–88.93%). The abundance of *Aerococcus* spp., the dominant genus, also increased significantly (41.58–54.75%) in the early fermentation stage (7 d). As fermentation progressed, the microbial structure of the PP treatment displayed specific differentiation. By 60 d, the abundance of *Atopostipes* spp. (33.24%) surpassed that of *Aerococcus* spp., establishing a symbiotic multi-genus system [8,33]. By contrast, *Aerococcus* spp. remained dominant in the LG and LP treatments (21.75–21.19%).

*Aerococcus* spp. were negatively correlated with NH_3_-N/TN in this study, indicating that they may reduce ammonia nitrogen accumulation by inhibiting the activity of proteolytic bacteria [34]. Notably, this finding contrasts with previous research concluding that *Aerococcus* spp. promote NH_3_-N/TN production in silage [13], which may be attributed to functional differences between strains or the presence of specific antibacterial components in nettle [35,36]. LEfSe analysis further revealed that the inoculation treatment accelerated the acidification process by enriching homolactic acid bacteria (such as *Pediococcus* spp. and *Enterococcus* spp.) [37]. Among them, *Lactobacillus* spp. in the PP treatment were significantly positively correlated with AA accumulation and inhibited the proliferation of *Clostridium_sensu_stricto_15* spp. [18,21]. The abundance of *Clostridia* spp. in the CK treatment reached 9.15%, whereas it remained below 1% in the inoculated treatments.

However, raw nettle materials possess a high buffering capacity and contain antibacterial substances, such as flavonoids, which limit the sustained efficacy of LAB [5,38]. Although the inoculation treatment significantly reduced the pH and increased the LA content in the initial stage (peaking at 30 d), LA was subsequently decomposed by *Clostridia* spp. because of insufficient acidity in the later stage [20]. This was evidenced by the significant increase in the BA content in the CK treatment and the failure of unnamed Lactobacillus to maintain acid production. In addition, although *Enterococcus* spp. were negatively correlated with NH_3_-N/TN, their acid-producing ability was inhibited by water-soluble extracts of nettle [39,40], further confirming that the antimicrobial activity of the raw material poses a substantial challenge to microbial regulation.

In summary, inoculation with LAB can effectively improve the nutritional retention and fermentation stability of nettle silage by regulating the microbial community structure (Firmicutes exceeding 80%), accelerating acidification, and inhibiting the metabolism of harmful bacteria [9]. However, the intrinsic characteristics of raw nettle materials—particularly their high pH and strong antibacterial activity—remain critical factors limiting fermentation efficiency.

## 5. Conclusions

This study confirmed that inoculation with LAB derived from *Urtica cannabinae* can effectively improve the quality of nettle silage by delaying the loss of DM and CP and inhibiting the degradation of NDF and ADF. In addition, the inoculation treatment increased the abundance of Firmicutes and *Aerococcus* spp. and decreased the accumulation of NH_3_-N/TN. In the PP treatment, the later-stage dominance of *Atopostipes* spp. and *Lactobacillus* spp. contributed to AA accumulation, which significantly inhibited the proliferation of *Clostridia* spp. However, the characteristics of raw nettle materials limit continuous acid production by LAB. Future efforts should focus on pretreatment or optimization of compound microbial inoculants to overcome this bottleneck.

## Figures and Tables

**Table 1:** Nutritional quality of nettle (*Urtica cannabina*) silage.

Index	Time	Treatment				*p*-Values		
		CK	LG	PP	LP	Treatment	Time	Treatment × Time
DM	7 d	37.72 ± 0.29 ^Aa^	38.74 ± 0.13 ^Aa^	38.63 ± 0.32 ^Aa^	38.64 ± 0.32 ^Aa^	0.001	0.000	0.909
	15 d	36.68 ± 0.31 ^Bab^	37.58 ± 0.52 ^ABb^	37.27 ± 0.58 ^ABb^	37.86 ± 0.21 ^Aa^			
	30 d	36.33 ± 0.25 ^Abc^	37.12 ± 0.46 ^Ab^	36.89 ± 1.4 ^Ab^	36.67 ± 0.53 ^Ab^			
	60 d	35.45 ± 0.52 ^Ac^	36.52 ± 0.39 ^Ab^	36.38 ± 0.59 ^Ab^	36.1 ± 0.16 ^Ab^			
CP (% DM)	7 d	16.23 ± 0 ^Aa^	16.08 ± 0.06 ^ABa^	15.95 ± 0.02 ^Ba^	16.08 ± 0.13 ^ABa^	0.000	0.000	0.000
	15 d	15.82 ± 0.04 ^BCb^	16 ± 0.03 ^Aa^	15.93 ± 0.15 ^ABa^	15.69 ± 0.21 ^Cb^			
	30 d	15.27 ± 0.01 ^Ac^	15.19 ± 0.02 ^Ab^	15.14 ± 0 ^Ab^	14.21 ± 0.08 ^Bc^			
	60 d	13.27 ± 0.01 ^Ad^	13.23 ± 0.03 ^Ac^	12.37 ± 0.01 ^Bc^	13.11 ± 0.03 ^Ad^			
NDF (% DM)	7 d	28.03 ± 1.18 ^Aa^	27.81 ± 1.36 ^Aa^	27.97 ± 0.43 ^Aa^	28.22 ± 1.17 ^Aa^	0.597	0.000	0.133
	15 d	27.45 ± 0.84 ^Aa^	27.14 ± 1.36 ^Aab^	27.43 ± 2.54 ^Aab^	27.41 ± 2.78 ^Aab^			
	30 d	27.77 ± 2.64 ^Aa^	26.57 ± 1.01 ^Bab^	26.35 ± 0.92 ^Bb^	26.47 ± 2.4 ^Bb^			
	60 d	25.59 ± 0.9 ^Bb^	26.22 ± 2.84 ^Ab^	26.31 ± 0.16 ^Ab^	26.46 ± 1.55 ^Ab^			
ADF (% DM)	7 d	19 ± 1.36 ^Aa^	19.01 ± 0.63 ^Aa^	19.16 ± 0.45 ^Aa^	19.24 ± 0.79 ^Aa^	0.014	0.000	0.351
	15 d	18.11 ± 0.32 ^Aa^	18.37 ± 0.71 ^Aa^	18.98 ± 0.84 ^Aab^	18.63 ± 1.34 ^Aab^			
	30 d	17.51 ± 1.88 ^Aa^	17.73 ± 0.9 ^Aa^	18.19 ± 0.58 ^Aab^	18.08 ± 0.78 ^Aab^			
	60 d	15.7 ± 0.11 ^Bb^	17.78 ± 1.89 ^Aa^	17.61 ± 0.17 ^Ab^	17.63 ± 1.14 ^Ab^			
EE (% DM)	7 d	6.26 ± 0.25 ^Aa^	6.94 ± 0.34 ^Aab^	6.59 ± 0.55 ^Aab^	7.04 ± 0.23 ^Aa^	0.006	0.000	0.014
	15 d	6.93 ± 0.87 ^Aa^	7.07 ± 0.81 ^Aa^	7.59 ± 0.6 ^Aa^	7.74 ± 0.42 ^Aa^			
	30 d	6.44 ± 0.44 ^Ba^	5.81 ± 0.97 ^Bbc^	5.45 ± 0.57 ^Bbc^	7.63 ± 0.64 ^Aa^			
	60 d	4.07 ± 0.32 ^Ab^	4.79 ± 0.19 ^Ac^	4.51 ± 0.18 ^Ac^	4.39 ± 0.15 ^Ab^			
WSCs (% DM)	7 d	4.92 ± 0.45 ^Aa^	4.8 ± 0.3 ^Aa^	4.83 ± 0.1 ^Aa^	4.74 ± 0.03 ^Aa^	0.908	0.000	0.998
	15 d	3.61 ± 0.11 ^Ab^	3.6 ± 0.24 ^Ab^	3.58 ± 0.19 ^Ab^	3.59 ± 0.09 ^Ab^			
	30 d	2.69 ± 0.01 ^Ac^	2.67 ± 0.03 ^Ac^	2.67 ± 0.03 ^Ac^	2.69 ± 0.11 ^Ac^			
	60 d	1.69 ± 0.07 ^Ad^	1.7 ± 0.13 ^Ad^	1.74 ± 0.16 ^Ad^	1.67 ± 0.12 ^Ad^			

Note: Capital letters indicate the same time but significant differences among different treatments (*p* < 0.05). Lowercase letters indicate the same treatment but significant differences between different times (*p* < 0.05). The same or missing letters indicate no statistically significant difference (*p* > 0.05). CK, control treatment; LG, inoculation with *Lactococcus garvieae*; PP, inoculation with *Pediococcus pentosaceus*; LP, co-inoculation with *Lactococcus garvieae* and *Pediococcus pentosaceus*. DM: dry matter; CP: crude protein; NDF: neutral detergent fiber; ADF: acid detergent fiber; EE: ether extract; WSCs: water-soluble carbohydrates.

**Table 2:** Fermentation quality of nettle (*Urtica cannabina*) silage.

Index	Time	Treatment				*p*-Values		
		CK	LG	PP	LP	Treatment	Time	Treatment × Time
pH	7 d	7.85 ± 0.03 ^Ab^	7.43 ± 0.02 ^Ba^	7.46 ± 0.02 ^Ba^	7.53 ± 0.03 ^Ba^	0.000	0.000	0.000
	15 d	7.77 ± 0.12 ^Ab^	7.07 ± 0.03 ^Cb^	7.14 ± 0.08 ^Cb^	7.29 ± 0.05 ^Bb^			
	30 d	8.35 ± 0.04 ^Aa^	6.35 ± 0.06 ^Cc^	6.42 ± 0.04 ^BCc^	6.56 ± 0.03 ^Bc^			
	60 d	8.47 ± 0.17 ^Aa^	5.01 ± 0.04 ^Bd^	5.24 ± 0.05 ^Cd^	5.78 ± 0.02 ^Dd^			
LA (g/kg DM)	7 d	2.68 ± 0.35 ^Ab^	3.02 ± 0.15 ^Ab^	2.8 ± 0.12 ^Ab^	3.06 ± 0.06 ^Aa^	0.000	0.000	0.000
	15 d	3.47 ± 0.07 ^ABa^	3.58 ± 0.54 ^Aab^	3.47 ± 0.07 ^ABa^	2.89 ± 0.06 ^Ba^			
	30 d	1.39 ± 0.15 ^Bc^	3.64 ± 0.71 ^Aa^	3.54 ± 0.06 ^Aa^	3.27 ± 0.07 ^Aa^			
	60 d	0.25 ± 0.01 ^Bd^	3.68 ± 0.37 ^Aa^	3.62 ± 0.17 ^Aa^	3.47 ± 0.15 ^Aa^			
AA (g/kg DM)	7 d	1.86 ± 0.06 ^Aa^	1.49 ± 0.04 ^Cc^	1.49 ± 0.08 ^Cc^	1.61 ± 0.01 ^Ba^	0.000	0.000	0.000
	15 d	1.92 ± 0.03 ^Ca^	2.05 ± 0.01 ^Ba^	2.26 ± 0.02 ^Aa^	1.66 ± 0.03 ^Da^			
	30 d	0.94 ± 0.04 ^Cb^	1.79 ± 0.13 ^Ab^	1.66 ± 0.05 ^Bb^	1.61 ± 0.01 ^Ba^			
	60 d	0.63 ± 0.03 ^Bc^	1.6 ± 0.08 ^Ac^	1.6 ± 0.03 ^Abc^	1.63 ± 0.04 ^Aa^			
PA (g/kg DM)	7 d	0.06 ± 0.01 ^Ab^	0.06 ± 0.01 ^Ab^	0.04 ± 0 ^Ab^	0.05 ± 0.02 ^Aa^	0.348	0.000	0.005
	15 d	0.09 ± 0.01 ^Bb^	0.09 ± 0.03 ^Bb^	0.09 ± 0.01 ^Bab^	0.16 ± 0 ^Aa^			
	30 d	0.06 ± 0 ^Ab^	0.09 ± 0.02 ^Ab^	0.08 ± 0.01 ^Aab^	0.07 ± 0.02 ^Ab^			
	60 d	0.19 ± 0.05 ^Aa^	0.18 ± 0.01 ^ABa^	0.12 ± 0.01 ^Ba^	0.13 ± 0.08 ^Bb^			
BA (g/kg DM)	7 d	ND	ND	ND	ND	0.000	0.000	0.000
	15 d	ND	ND	ND	ND			
	30 d	3.3 ± 0.08 ^Ab^	2.09 ± 0.01 ^Cb^	2.8 ± 0.01 ^Bb^	3.04 ± 0.2 ^ABb^			
	60 d	5.41 ± 0.54 ^Aa^	3.31 ± 0.37 ^Ba^	3.36 ± 0.17 ^Ba^	3.68 ± 0.07 ^Ba^			
NH_3_-N/TN (%)	7 d	1.06 ± 0.06 ^ABc^	0.34 ± 0.19 ^Bc^	1.48 ± 0.44 ^Ab^	1.88 ± 0.1 ^Ac^	0.000	0.000	0.000
	15 d	1.91 ± 0.04 ^Ac^	0.81 ± 0.06 ^Bc^	1.31 ± 0.01 ^ABb^	1.49 ± 0.14 ^ABc^			
	30 d	6.71 ± 0.18 ^Ab^	4.4 ± 0.08 ^Bb^	5.04 ± 0.1 ^Ba^	5.16 ± 0.01 ^Bb^			
	60 d	9.04 ± 0.19 ^Aa^	5.79 ± 0.11 ^Ba^	6.13 ± 0.29 ^Ba^	6.78 ± 1.87 ^Ba^			

Note: Capital letters indicate the same time but significant differences among different treatments (*p* < 0.05). Lowercase letters indicate the same treatment but significant differences between different times (*p* < 0.05). The same or missing letters indicate no statistically significant difference (*p* > 0.05). CK, control treatment; LG, inoculation with *Lactococcus garvieae*; PP, inoculation with *Pediococcus pentosaceus*; LP, co-inoculation with *Lactococcus garvieae* and *Pediococcus pentosaceus*. LA: lactic acid; AA: acetic acid; PA: propionic acid; NH_3_-N/TN: ammonia nitrogen to total nitrogen ratio. ND: not detected. The following table shows the same results.

## Data Availability

The data that support this study are available in this article. The raw data from this study are available upon request from the corresponding author. Data related to the bacterial community in the Sequence Read Archive (SRA) can be found at the National Center for Biotechnology Information (NCBI) (SRA number: PRJNA1266637).

## Abbreviations

The following abbreviations are used in this manuscript:

LAB	Lactic acid bacteria
WSC	Water-soluble carbohydrate
CK	Control group
DM	Dry matter
CP	Crude protein
NDF	Neutral detergent fiber
ADF	Acid detergent fiber
EE	Ether extract
AA	Acetic acid
PA	Propionic acid
BA	Butyric acid
